# Real-time fluorescence-guided adhesiolysis with indocyanine green in intra-abdominal surgery (with video)

**DOI:** 10.1038/s41598-024-51450-8

**Published:** 2024-01-06

**Authors:** Qiangxing Chen, Yu Cai, Ke Cheng, Zixin Chen, Jun Li, Shangdi Wu, Bing Peng

**Affiliations:** 1 Division of Pancreatic Surgery, Department of General Surgery, West China Hospital of Sichuan University, Chengdu, 610041 China; 2 West China School of Medicine, West China Hospital of Sichuan University, Chengdu, China; 3 Division of Liver Surgery, Department of General Surgery, West China Hospital of Sichuan University, Chengdu, China; 4 Department of General Surgery, Nanchong Central Hospital, The Second Clinical College of North Sichuan Medical College, Nanchong, Sichuan China

**Keywords:** Gastroenterology, Medical research

## Abstract

Intra-abdominal adhesions have consistently posed a challenge for surgeons during procedures. This study aims to investigate the feasibility of utilizing indocyanine green (ICG) in conjunction with near-infrared imaging for the detection of intra-abdominal adhesions. In vitro, we analyzed factors affecting ICG fluorescence. We divided SD rats into groups to study ICG excretion in different digestive tract regions. Additionally, we reviewed surgical videos from previous cholecystectomy cases, categorizing them by ICG injection timing and assessing fluorescence imaging in various digestive tract regions. Finally, we preoperatively injected ICG into two cholecystectomized patients with abdominal adhesions, guiding intraoperative adhesiolysis with near-infrared fluorescence imaging. In vitro, we observed a significant influence of protein and ICG concentrations on ICG fluorescence intensity. Our rat experiments unveiled a strong and highly significant correlation (Kendall’s tau-b = 1, *P* < 0.001) between the timing of ICG injection and the farthest point of intestinal fluorescence. A retrospective case analysis further validated this finding (Kendall’s tau-b = 0.967, *P* < 0.001). Under the guidance of fluorescence navigation, two cholecystectomized patients with intra-abdominal adhesions successfully underwent adhesiolysis, and no postoperative complications occurred. The intraoperative combination of ICG with near-infrared fluorescence imaging effectively enhances the visibility of the liver, bile ducts, and various segments of the gastrointestinal tract while providing real-time navigation. This real-time fluorescence guidance has the potential to aid surgeons in the dissection of intra-abdominal adhesions.

## Introduction

Over 90% of abdominal surgeries result in intra-abdominal adhesions, commonly encountered by general surgeons, vascular surgeons, gynecologists, and urologists^1–3^. Adhesion formation has significant negative implications for patient health and is associated with increased clinical workload. Furthermore, adhesiolysis during subsequent surgeries is associated with an increased risk of inadvertent intestinal injury and prolonged surgical time^4^. Injury to the gastrointestinal tract can have serious consequences for the patient, and in some cases patients develop life-threatening complications such as sepsis following a leaky bowel.

Minimizing tissue damage or avoiding injury to critical anatomical structures during adhesion dissolution in patients with intra-abdominal adhesions is a recurring concern for general surgeons, gynecologists, and urologists. Nonetheless, there currently needs to be more literature addressing this matter. In a systematic review and meta-analysis conducted by Limperg et al.^5^, the diagnostic accuracy of ultrasound in detecting intra-abdominal adhesions was investigated. The study reveals that preoperative ultrasound assessment of visceral slide yields a notably high negative predictive value in identifying periumbilical intestinal adhesions among at-risk patients. This approach is a valuable tool for locating adhesion-free regions, thereby ensuring the safety of laparoscopic entry. However, its utility in guiding intraoperative adhesiolysis remains somewhat restricted.

**Figure 1:**
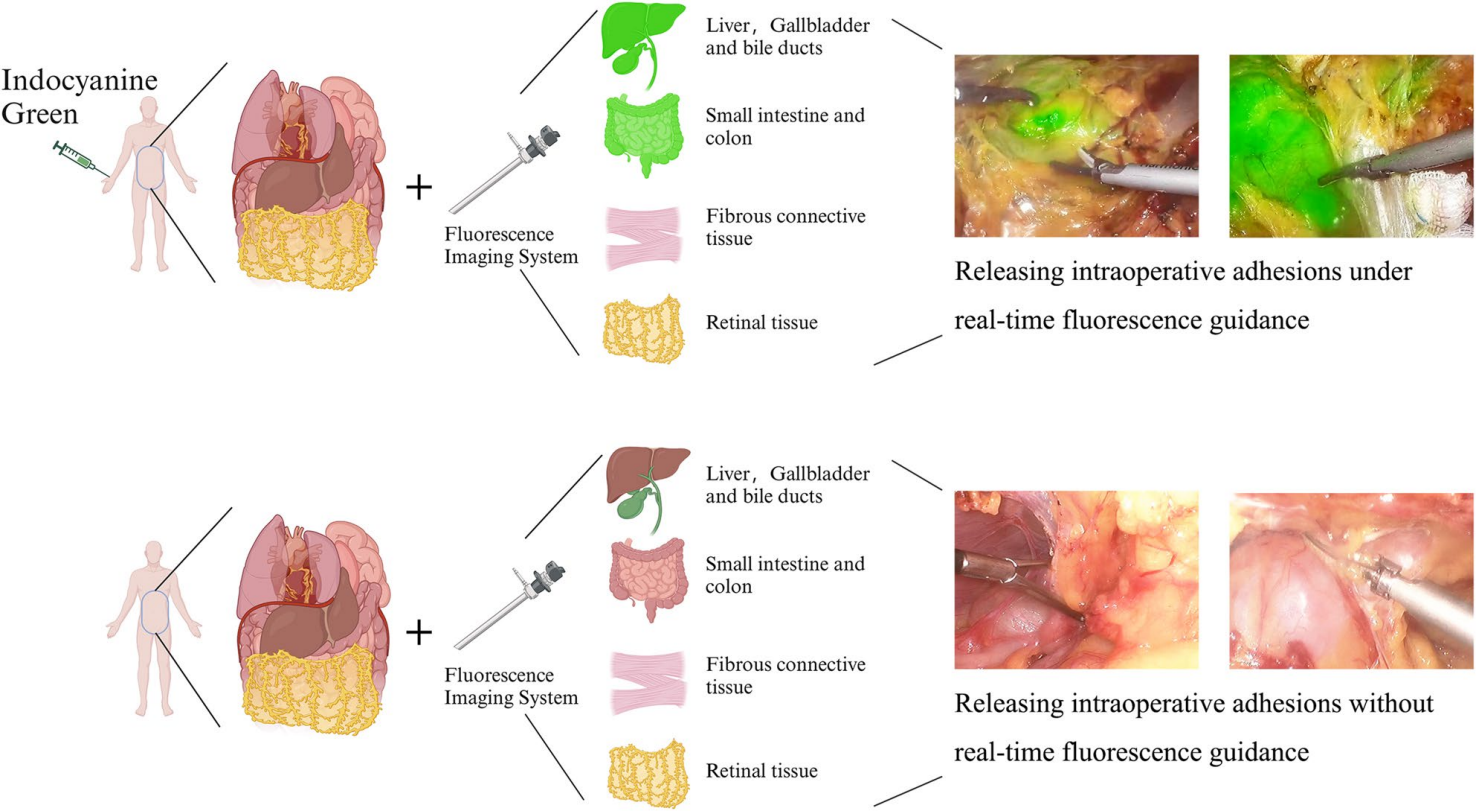
Application of ICG with near-infrared fluorescence imaging system for dissecting intra-abdominal adhesions. Prior to abdominal surgery, ICG is administered via peripheral vein injection. Intraoperatively, the near-infrared fluorescence imaging system is utilized to visualize tissues, enabling the release of intraoperative adhesions under real-time fluorescence guidance. *Note*: Following peripheral intravenous injection of a specific dose of ICG, due to the pharmacokinetic characteristics of ICG, it undergoes hepatic metabolism, is excreted into the gastrointestinal tract via the bile duct, and does not participate in enterohepatic circulation. Additionally, after binding to proteins, ICG, when excited by near-infrared light, emits light at a longer wavelength, approximately 830 nm.

Recently, indocyanine green (ICG) combined with a near-infrared fluorescence imaging system has been applied in various surgical scenarios, including liver resection^6,7^, cholecystectomy^8^, and assessment of gastric and intestinal anastomosis blood supply^9^. In these scenarios, the ICG combined with the near-infrared fluorescence imaging system has played a crucial role in real-time guidance, significantly assisting surgeons in their decision-making. However, the applicability of ICG combined with near-infrared fluorescence imaging system for adhesiolysis in abdominal adhesion cases is a question of concern. It is known that after intravenous injection, ICG is metabolized by hepatocytes and excreted into the bile almost entirely^10,11^. Additionally, when stimulated by near-infrared light (700–900 nm) after ICG injection, light with a wavelength of approximately 830 nm is emitted. In our previous studies^8^, we observed varying degrees of biliary fluorescence imaging intensity after injection of the same dose of ICG at different time points, suggesting that the excreted ICG does not participate in the enterohepatic circulation. In humans, a specific dose of ICG (0.5 mg/Kg) is injected via a peripheral vein, and approximately 30 min after injection, more than 98% of ICG is taken up by the liver^17^, and its in vivo clearance enters the hepatic elimination phase^18^. Building on our prior research into the excretion characteristics and fluorescence imaging properties of ICG, this study investigates the feasibility of employing ICG combined with a near-infrared fluorescence imaging system for intra-abdominal adhesiolysis during abdominal surgery. The study’s overarching objective is to provide real-time guidance for intraoperative adhesion release using fluorescence (Fig. 1, Created with BioRender.com), thereby minimizing the risk of tissue damage or injury to vital anatomical structures.

## Results

### The presence of protein in the liquid is necessary for ICG fluorescence imaging

**Figure 2:**
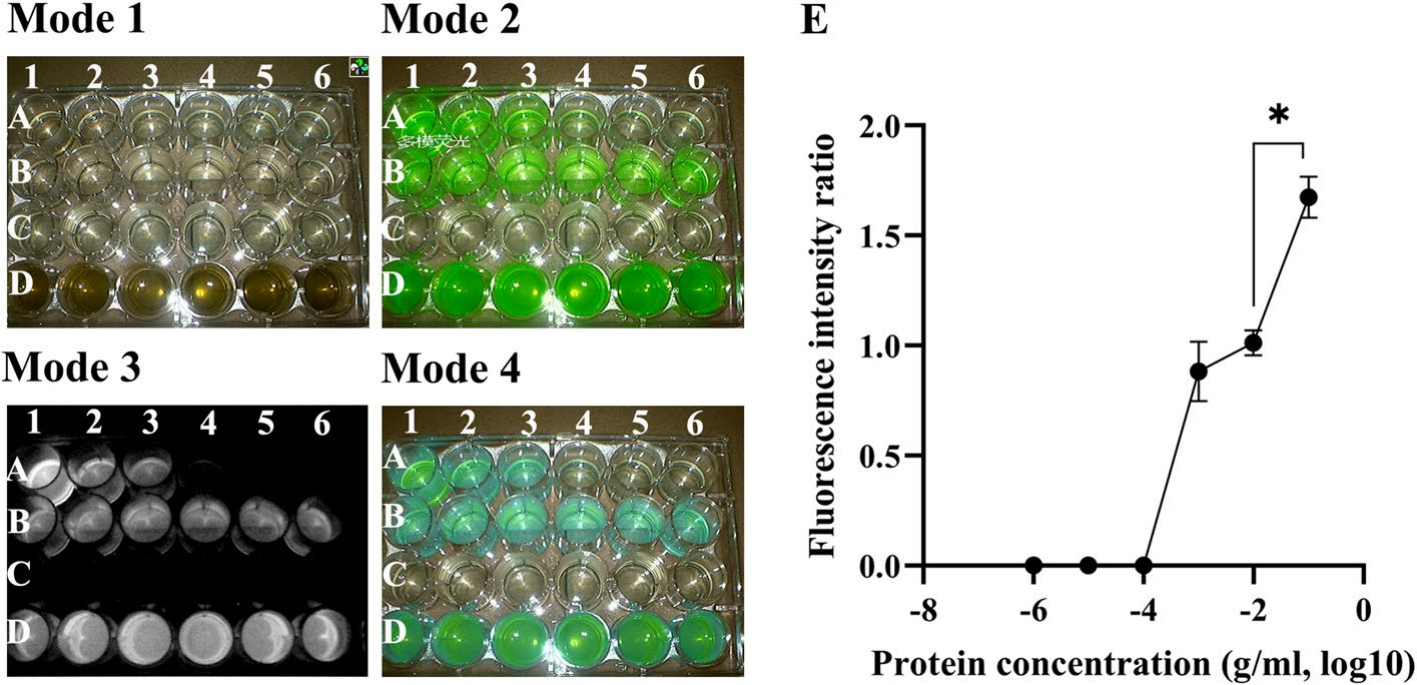
ICG fluorescence imaging conditions. Correlation analysis revealed a positive association between protein concentration and fluorescence intensity ratio (Kendall’s tau-b = 0.828, *P* < 0.0001) (**E**). The fluorescence intensity ratio of A1 group was significantly different from that of A2 group (*P* < 0.05) (**E**). Fluorescence intensity ratio: B was used as the reference well, and the fluorescence intensity ratio was calculated by comparing the fluorescence intensity of A with that of B. (**A**) a mixture of albumin and ICG (concentration: 0.01 mg/ml) with varying protein concentrations (A1: 0.1 g/ml, A2: 0.01 g/ml, A3: 0.001 g/ml, A4: 0.0001 g/ml, A5: 0.00001 g/ml, A6: 0.000001 g/ml). (**B**) a mixture of albumin (concentration: 0.01 g/ml) and ICG (concentration: 0.01 mg/ml). (**C**) a mixture of saline and ICG (concentration: 0.01 mg/ml). (**D**) a mixture of bile and ICG (concentration: 0.01 mg/ml).

From Mode 3 (Fig. 2), we observed that only the sodium chloride solution with ICG did not exhibit fluorescence under the fluorescence laparoscope (Fig. 2-Mode3-C). Significant fluorescence imaging was observed in the liquid containing a certain concentration of ICG and a certain protein concentration (Fig. 2-Mode3-B). In contrast, fluorescence imaging gradually weakened as the protein concentration decreased in the liquid (Fig. 2-Mode3-A,E). There was a strong positive correlation between protein concentration and fluorescence intensity, with Kendall’s tau-b = 0.828, *P* < 0.0001. Adding a certain concentration of ICG to the bile (Fig. 2-Mode3-D) also resulted in noticeable fluorescence imaging.

### Changes in ICG concentration affect the intensity of fluorescence imaging

**Figure 3:**
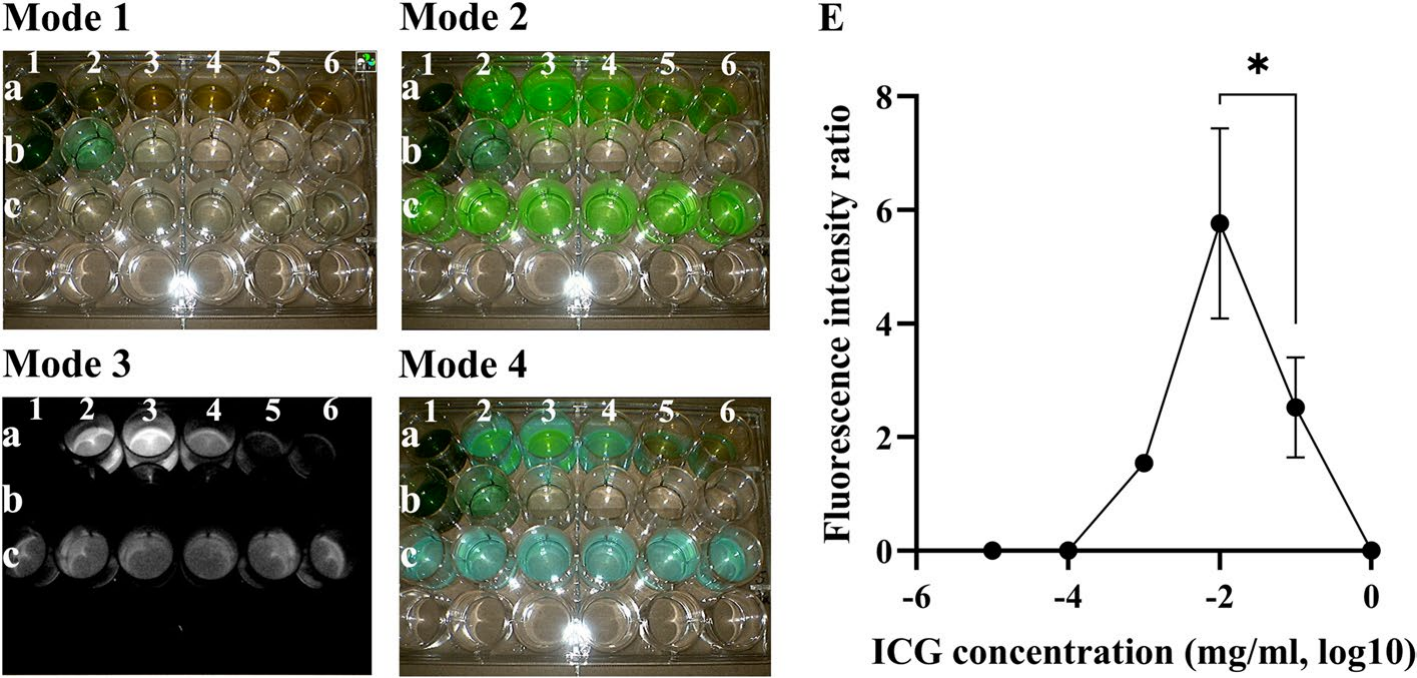
Fluorescence imaging required ICG concentration range. Comparison of fluorescence intensity ratio between a3 and a2 wells showed statistically significant differences (*P* < 0.05). Fluorescence intensity ratio: Group c was used as the reference well, and the fluorescence intensity ratio was calculated by comparing the fluorescence intensity of Group a with that of Group c. Group a: Mixed liquid containing ICG and bile (ICG concentration: a1 = 1 mg/ml, a2 = 0.1 mg/ml, a3 = 0.01 mg/ml, a4 = 0.001 mg/ml, a5 = 0.0001 mg/ml, a6 = 0.00001 mg/ml). Group b: Physiological saline containing ICG (ICG concentration: 0.01 mg/ml). Group c: Mixed liquid containing ICG (concentration: 0.01 mg/ml) and albumin (protein concentration: 0.01 g/ml). ICG concentration (0.00001 mg/ml to 0.1 mg/ml) showed a positive correlation with the fluorescence intensity ratio (Kendall’s tau-b = 0.70, *P* = 0.001).

It can be observed that the sodium chloride solution with different concentrations of ICG did not exhibit fluorescence imaging (Fig. 3-Mode3-b). As the control group, the protein solution with a certain concentration of ICG showed fluorescence imaging (Fig. 3-Mode3-c). The liquid containing different concentrations of ICG prepared with bile (a1 = 1 mg/ml, a2 = 0.1 mg/ml, a3 = 0.01 mg/ml, a4 = 0.001 mg/ml, a5 = 0.0001 mg/ml, a6 = 0.00001 mg/ml) displayed varying intensities of fluorescence imaging (Fig. 3-Mode3-a). The optimal ICG concentration produced the maximum fluorescence imaging effect (Fig. 3-Mode3-a3,E). Moreover, within the range of 0.00001 mg/ml to 0.1 mg/ml, there was a strong positive correlation between ICG concentration and fluorescence intensity, with Kendall’s tau-b = 0.70, *P* = 0.001 (Fig. 3E).

### Fluorescence visualization of intestinal segments following jugular vein injection of ICG in rats at different time points

**Figure 4:**
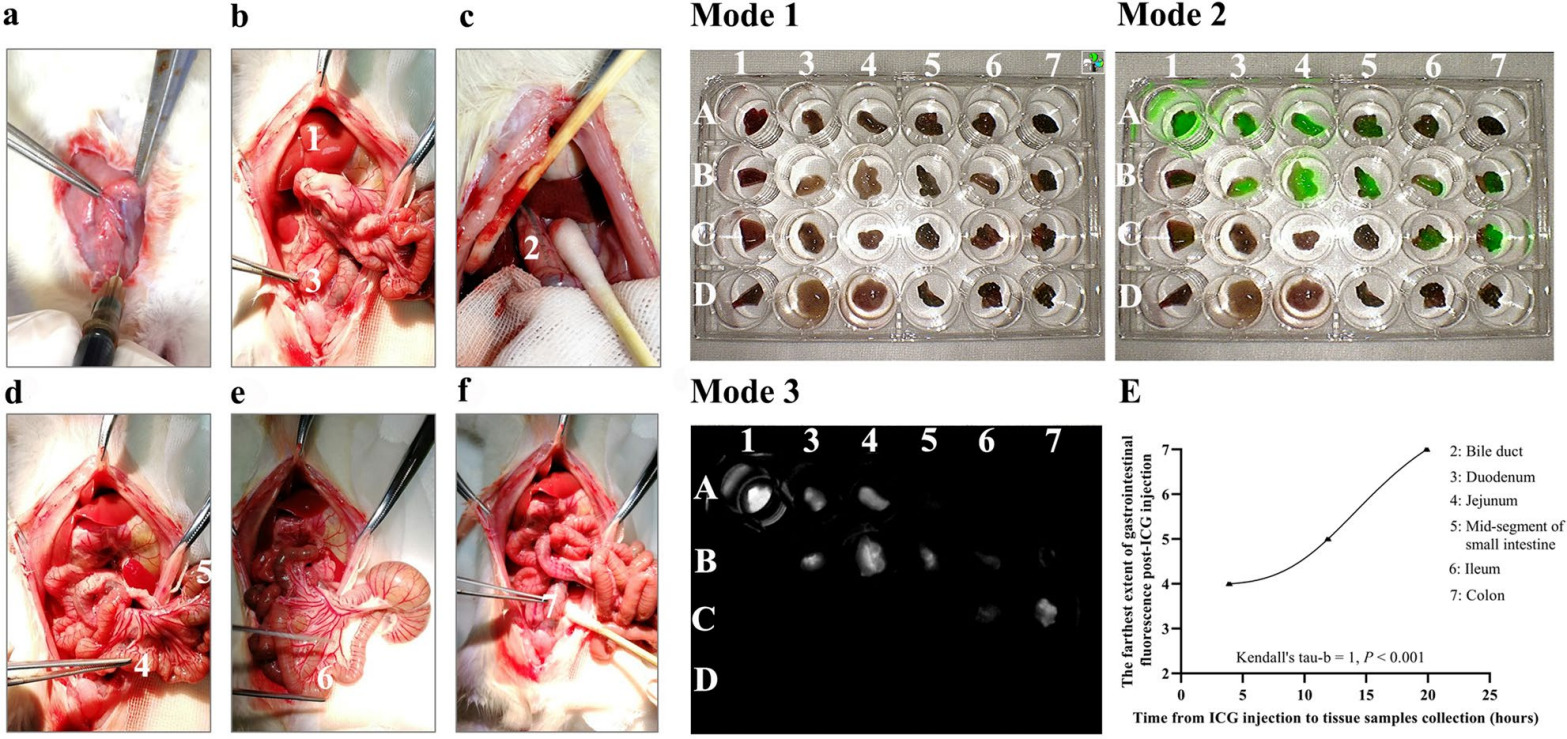
Correlation analysis of fluorescence imaging at different sites of the gastrointestinal tract and ICG injection time. (**a**) Rats were injected with 1 ml ICG (concentration: 10 mg/ml) through the jugular vein. (**b**–**f**) Tissue samples were obtained after ICG injection: 1. Liver; 2. Bile duct; 3. Duodenum; 4. Jejunum; 5. Mid-segment of small intestine; 6. Ileum; 7. Colon. A: Rats Groups A. B: Rats Groups B. C: Rats Groups C. D: Rats Groups D. Tissue samples were collected at 4 h post-ICG injection for Groups A and D, 12 h for Group B, and 20 h for Group D. E: Correlation analysis between the farthest extent of gastrointestinal fluorescence after ICG injection and the time from ICG injection to tissue samples collection.

After injecting 10 mg of ICG into the rat’s jugular vein (Fig. 4a), we collected tissues from the liver (Fig. 4b, Mode 1–1), duodenum (Fig. 4b, Mode 1–3), proximal small intestine (Fig. 4d, Mode 1–4), mid-segment of small intestine (Fig. 4d, Mode 1–5), distal small intestine (Fig. 4e, Mode 1–6), and colon (Fig. 4f, Mode 1–7) at three different time points for fluorescence imaging. In Group A (Fig. 4, Mode 1-A) and the saline injection group (Group D, Fig. 4, Mode 1-D), we collected tissues 4 h post-injection. In Group D, no fluorescence was observed in any tissue (Fig. 4, Mode 3-D), while in Group A, fluorescence was visible in the liver, duodenum, and proximal small intestine (Fig. 4, Mode 3-A). In Group B, 12 h after ICG injection, fluorescence was observed from the duodenum to the mid-small intestine (Fig. 4, Mode 3-B). In Group C, 20 h after ICG injection, fluorescence was observed in the distal small intestine and colon (Fig. 4, Mode 3-C). There was a strong positive correlation between the time from ICG injection to tissue samples collection and the farthest point of fluorescence visualization in the digestive tract, with Kendall’s tau-b = 1, *P* < 0.001 (Fig. 4E).

### Fluorescence visualization of intestinal segments following peripheral vein injection of ICG in patients at different time points

**Figure 5:**
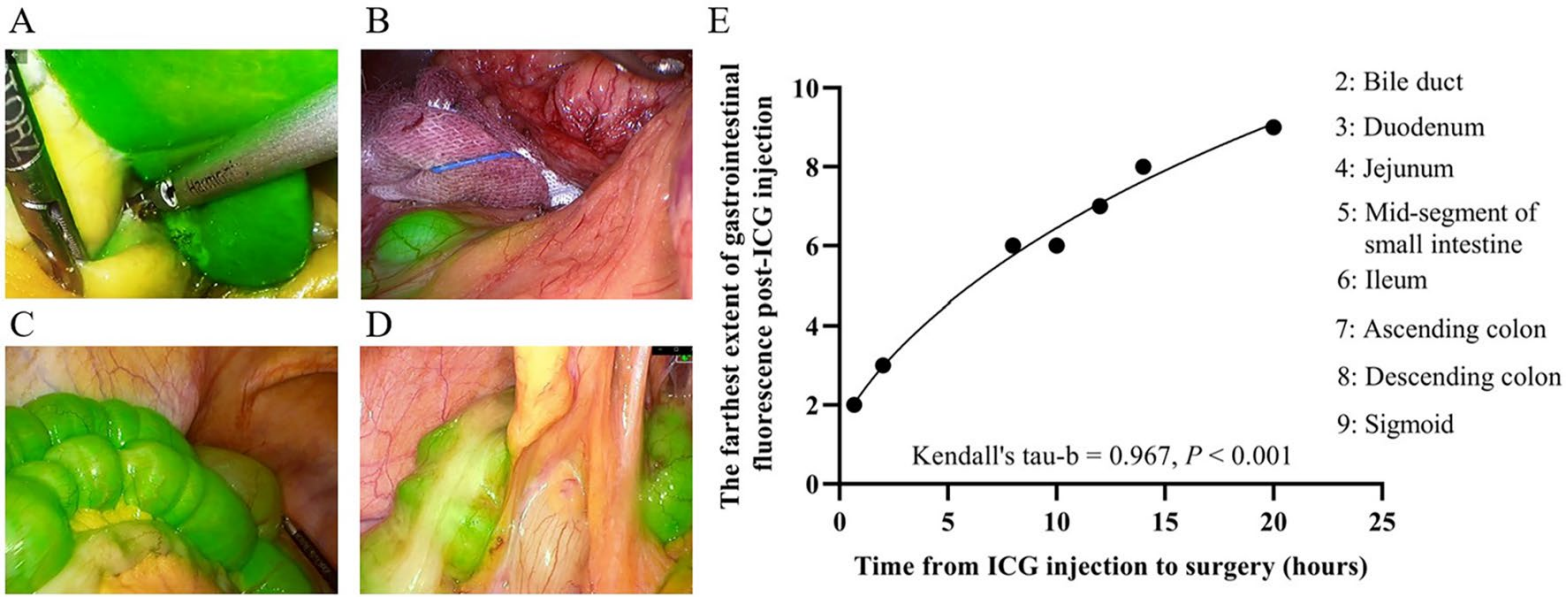
Fluorescence Imaging of Different Gastrointestinal Sites in the Human Digestive Tract Following ICG Injection at Different Time Points. (**A**) We observed that the liver exhibited fluorescence immediately after ICG injection for 40 min. (**B**) The duodenum showed fluorescence at 2 h after ICG injection. (**C**) The small intestine showed fluorescence from 8 to 14 h after ICG injection. (**D**) The colon exhibited fluorescence at 20 h after ICG injection. (**E**) Correlation analysis between the farthest extent of gastrointestinal fluorescence after ICG injection and the time from ICG injection to surgery.

We retrospectively reviewed 36 videos of cholecystectomy procedures conducted using ICG-assisted near-infrared fluorescence imaging. Prior to surgery, all patients received intravenous injection of 10 mg ICG. Among them, 3 patients were injected with ICG approximately 40 min before surgery, 4 patients received the injection 2 h prior, 12 patients received the injection 8–10 h before surgery, 12 patients received the injection 12–14 h before surgery, and 5 patients received the injection 20 h before surgery. We examined the intraoperative fluorescence imaging of the liver and gastrointestinal tract. We observed that the liver exhibited fluorescence immediately after ICG injection for 40 min (Fig. 5A), the duodenum showed fluorescence at 2 h after ICG injection (Fig. 5B), the small intestine showed fluorescence from 8 to 14 h after ICG injection (Fig. 5C), and the colon exhibited fluorescence at 20 h after ICG injection (Fig. 5D). Similar to the findings in the rat study, there was a significant positive correlation between the time from ICG injection to surgery and the farthest segment of fluorescence visualization, with Kendall’s tau-b = 0.967, *P* < 0.001 (Fig. 5E).

### Real-time navigation of adhesion release during surgery using ICG in patients with abdominal adhesions

**Figure 6:**
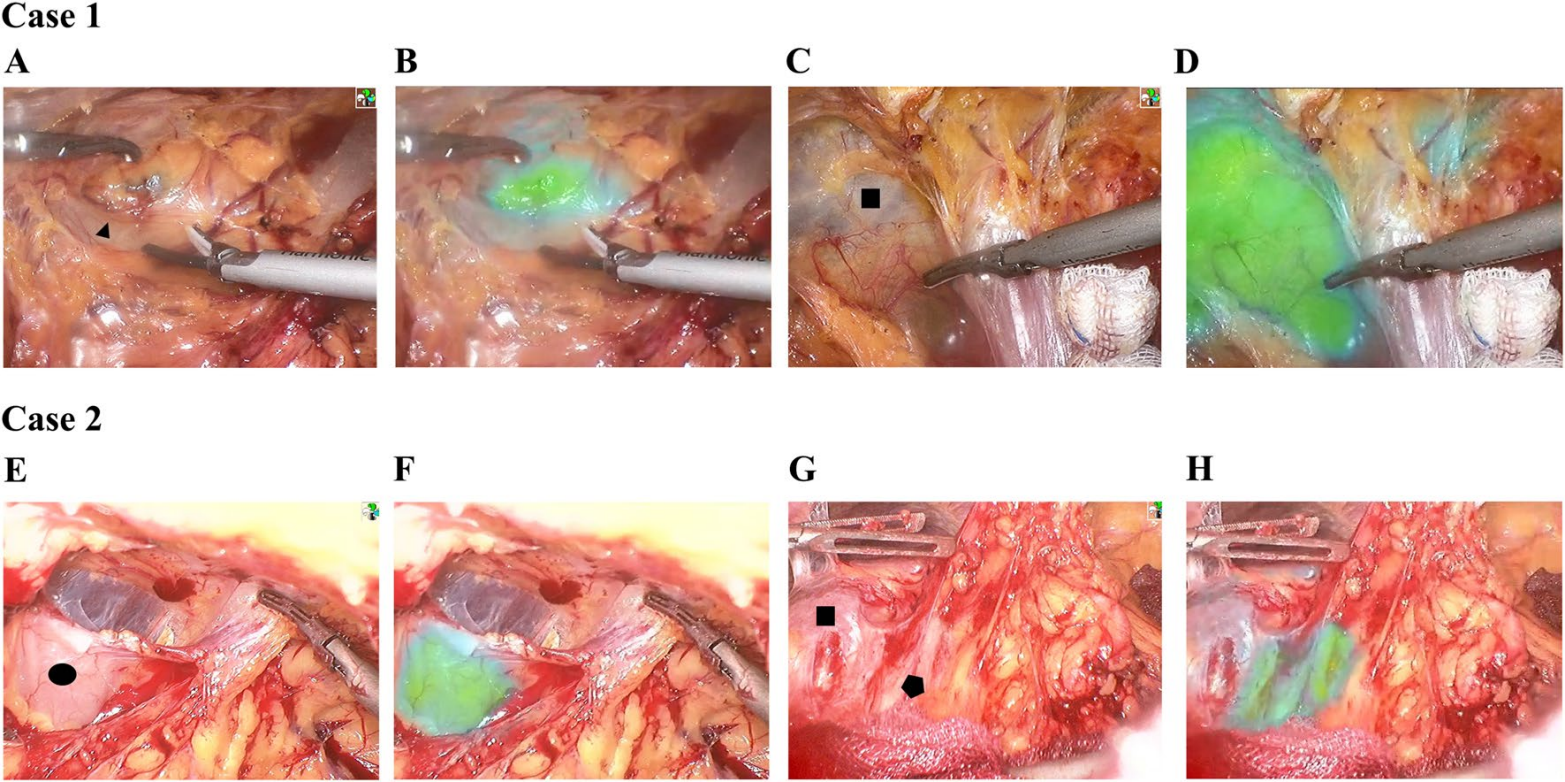
Real-time navigation during intraoperative adhesiolysis using ICG-Integrated Near-Infrared Fluorescence Imaging System. (**A**–**D**) Dissection of intra-abdominal adhesions under real-time fluorescence navigation, revealing the liver and gastric tissues in Patient A. (**E**–**H**) Dissection of intra-abdominal adhesions under real-time fluorescence navigation, exposing the liver, small intestine, and transverse colon tissues in Patient. ▲: Stomach; ■: Liver; ⬬: Small intestine; ⬣: transverse colon.

Based on the results of intestinal fluorescence imaging after ICG excretion in the body, we applied a “two-step” ICG injection strategy for these two cholecystectomized patients, administering 1 ml of ICG (concentration: 10 mg/ml) at 20 h and 4 h before surgery, respectively. Subsequently, intraoperative fluorescence laparoscopy was utilized for real-time navigation. Significant adhesions were observed in the patient’s upper abdomen (Fig. 6A,C,E,G). We gradually released the abdominal adhesions with real-time fluorescence navigation (Video) while avoiding the fluorescent areas, revealing the liver, stomach, small intestine, and transverse colon (Fig. 6B,D,F,H). With the assistance of real-time fluorescence navigation, we successfully conducted a common bile duct exploration and removal of the bile duct stone in Case 1. Likewise, in Case 2, we managed to remove residual gallbladder stones. During the follow-up period, both patients experienced smooth recoveries, with no postoperative leaks, and were discharged in good condition (Supplementary Video S1).

## Discussion

The dissection of intra-abdominal adhesions is a challenge faced by every abdominal surgeon^12^, as severe adhesions can result in inadvertent organ injuries during the process. Despite various methods available for the prevention and treatment of postoperative intra-abdominal adhesions^3,13–16^, there is a relative paucity of research reports focusing on the safe and efficient dissection of these adhesions.

This study explored the feasibility of using ICG combined with near-infrared fluorescence imaging system for intra-abdominal adhesion dissection. Our in vitro experiments confirmed the need for appropriate ICG and protein concentrations for efficient fluorescence imaging. Through experiments in both rats and humans, we verified that ICG, upon injection, could be excreted into the gastrointestinal tract and produce fluorescent images of different segments of the intestine. In the rat experiment, we observed a significant positive correlation between the ICG injection time and the intestine’s furthest segment showing fluorescence imaging. Further clinical observations also confirmed this association. Moreover, we found that injecting ICG too early before surgery failed to produce fluorescence imaging of the duodenum and proximal small intestine. Conversely, injecting ICG too late before surgery resulted in a lack of fluorescence imaging in the colon region. These research findings provide a theoretical basis for applying ICG in intra-abdominal adhesion dissection. In order to maximize the display of the imaged gastrointestinal area, we creatively adopted a “two-step” method of ICG injection for a patient with intra-abdominal adhesions, administering 10 mg ICG at 20 h and 4 h before surgery. During the dissection of intra-abdominal adhesions, we accomplished the release of adhesions in two cholecystectomized patients with severe intra-abdominal adhesions, utilizing real-time navigation provided by the fluorescence imaging system.

Our research results are inspiring, presenting a practical approach to visualize most gastrointestinal tracts. This approach enhances the surgeon’s identification of the gastrointestinal tract, thereby avoiding inadvertent damage during intraoperative adhesiolysis. Similar to our findings, Lee et al. injected 10 ml of ICG above and below the narrowed portion of the diseased ureter through a ureteral catheter or percutaneous nephrostomy tube. During surgery, NIRF was activated to aid in identifying the ureter and locating the margins of ureteral stenosis. This technique enables rapid and accurate identification of the ureter and precise localization of ureteral stenosis^19^. Building on this technology, Guan et al. recently published a case report where they successfully performed surgery for endometriosis with the assistance of this technique, avoiding inadvertent ureteral damage during surgery^20^. Furthermore, Nitta et al. used the IRIS U kit for indocyanine green injection into the urethra during low anterior resection of total mesorectal excision (TME) for rectal cancer, followed by visualization under NIR. Researchers could easily detect and visualize the IRIS urethral kit. The use of the infrared illumination system, the urethral kit (IRIS U kit), allowed real-time identification of the prostatic segment of the urethra. This TaTME approach brings new perspectives in avoiding iatrogenic urethral injury^21^. We believe that the intraoperative application of ICG for real-time navigation will become more widespread in the future.

ICG is safety. The United States Food and Drug Administration recommends using ICG for various purposes, including the visualization of vessels, blood flow, tissue perfusion, extrahepatic biliary ducts, and lymph nodes during lymphatic mapping for cervical and uterine tumors (P2). Additionally, it is essential to note that no toxicity findings, including hematological, clinical chemistry, and histopathological assessments, were reported either one or fourteen days following intravenous administration of ICG at a dose of 20 mg/kg, which is considered the no observed adverse effect level (P33, P74) (https://www.fda.gov/media/124114/download).

While the number of patients included in this study for adhesion dissection was limited, our research systematically explores the theoretical foundation and practical feasibility of this technique both in vitro and in vivo. It offers a promising approach for potential patients with intra-abdominal adhesions requiring surgery while also providing valuable guidance for general surgeons, gynecologists, and urologists in their decision-making during adhesion dissection. It is important to note that, based on our research findings and the pharmacokinetic characteristics of ICG after peripheral intravenous administration, we believe that our proposed method may be more suitable for patients with a patent gastrointestinal tract and suspected intra-abdominal adhesions requiring surgery. However, it is not suitable for patients with concurrent intestinal obstruction and adhesions, as ICG delivery to the intestines may be compromised. Therefore, when the operating surgeon suspects the potential for inadvertent gastrointestinal injury during adhesion dissection, we recommend using ICG injection combined with fluorescence imaging to enhance intraoperative identification of the gastrointestinal tract for separating intra-abdominal adhesions. We should recognize that pioneering techniques like this may not be universally applicable. Furthermore, future prospective randomized controlled trials will further validate the utility of this approach.

## Conclusions

The intraoperative combination of ICG with near-infrared fluorescence imaging effectively enhances the visibility of the liver, bile ducts, and various segments of the gastrointestinal tract while providing real-time navigation. This real-time fluorescence guidance has the potential to aid surgeons in the dissection of intra-abdominal adhesions.

## Methods

### Ethics declarations

This study approved by the Animal Care and Use Committee of Sichuan University and Biomedical Research Ethics Committee of West China Hospital of Sichuan University. Written informed consent was obtained from this study participant. Our study is reported in accordance with ARRIVE guidelines (https://arriveguidelines.org). We confirmed that all methods were carried out in accordance with relevant guidelines and regulations, and we confirmed that all experimental protocols were approved by the Animal Care and Use Committee of Sichuan University and the Biomedical Research Ethics Committee of West China Hospital of Sichuan University.

### The near-infrared fluorescence imaging system

Fluorescence imaging was performed using a high-definition fluorescence laparoscope (OptoMedic Endoscopes, China) inserted through a standard 12 mm subumbilical trocar port, as described in previous studies (reference). The imaging system was equipped with a visible and near-infrared (NIR) light source to facilitate fluorescence detection. Moreover, the NIR fluorescence (NIRF) imaging system offered four distinct modes: mode 1 utilized white light imaging alone, mode 2 combined green fluorescence imaging with white light imaging, mode 3 focused on original fluorescence imaging without white light imaging, and mode 4 involved fluorescence and white light imaging after pseudo-color processing. These different modes allowed for enhanced visualization and improved identification of target tissues during the procedure.

### ICG fluorescence imaging conditions

To explore the conditions for ICG fluorescence imaging, we conducted in vitro experiments. We prepared the following liquid solutions: A) albumin solutions containing ICG (concentration: 0.01 mg/ml) with varying albumin concentrations (0.1 g/ml, 0.01 g/ml, 0.001 g/ml, 0.0001 g/ml, 0.00001 g/ml, 0.000001 g/ml); B) albumin solution containing ICG (concentration: 0.01 mg/ml) with albumin concentration of 0.01 g/ml; C) NaCl solution containing ICG (concentration: 0.01 mg/ml); and D) bile solution containing ICG (concentration: 0.01 mg/ml). Subsequently, the liquid solutions were imaged using a near-infrared fluorescence imaging system. The fluorescence intensity of each well in the fluorescent images was measured by Image-Pro Plus (IPP; produced by Media Cybernetics Corporation, USA). We analyzed the correlation between fluorescence intensity and protein concentration.

### ICG concentration range for fluorescence imaging

To explore the ICG concentration range required for fluorescence imaging, we prepared three liquid solutions containing ICG. Solution a: bile solution containing ICG (ICG concentrations of 1 mg/ml, 0.1 mg/ml, 0.01 mg/ml, 0.001 mg/ml, 0.0001 mg/ml, 0.00001 mg/ml). Solution b: sodium chloride solution containing ICG (ICG concentrations of 1 mg/ml, 0.1 mg/ml, 0.01 mg/ml, 0.001 mg/ml, 0.0001 mg/ml, 0.00001 mg/ml). Solution c: albumin solution containing ICG (albumin concentration: 0.01 g/ml; ICG concentration: 0.01 mg/ml). Liquid solutions were imaged using a near-infrared fluorescence imaging system. We analyzed the correlation between fluorescence intensity and ICG concentration.

### Excretion of ICG in the rat digestive tract

Following ICG injection, its excretion occurs via bile. Our previous study^8^ observed noticeable fluorescence imaging in the bile collected from patients with T-tubes two hours after the intravenous administration of 10 mg ICG. However, after 20 h, the collected bile showed reduced fluorescence imaging. To further explore the time required for ICG excretion in different parts of the intestinal tract, we randomly divided 12 SD rats into four groups (Groups A, B, C, and D), with three rats in each group. Animal experiments were carried out under the National Institutes of Health guidelines for the care and use of laboratory animals. Rats in Groups A, B, and C received a 1 ml ICG (concentration: 10 mg/ml) through intravenous injection, while rats in Group D received an equivalent volume of saline without ICG. Subsequently, the rats were sacrificed at different time points to obtain tissue samples from six locations: liver tissue, duodenum at the junction with the common bile duct, upper small intestine 10 cm from the ligament of Treitz, small intestine 20 cm from the ligament of Treitz, lower small intestine 5 cm from the ileocecal valve, and intestinal contents 5 cm from the anus. Tissue samples were collected at 4 h post-ICG injection for Groups A and D, 12 h for Group B, and 20 h for Group C. These tissue samples were processed using a near-infrared fluorescence imaging system. We assigned a numerical value to each digestive tract region based on its proximity (liver as 1, bile duct as 2, duodenum as 3, jejunum as 4, mid-segment of small intestine as 5, ileum as 6, and colon as 7). Subsequently, we conducted a correlation analysis between the time from ICG injection to tissue samples collection and the farthest imaging position in the digestive tract with fluorescence visualization.

### Excretion of ICG in the human digestive tract

Based on the fluorescence imaging results of ICG excretion in the rat digestive tract, we categorized and reviewed surgical videos of previous cholecystectomy cases that combined ICG with near-infrared fluorescence imaging, according to the timing of ICG injection. We assigned a numerical value to each digestive tract region based on its proximity (liver as 1, bile duct as 2, duodenum as 3, jejunum as 4, mid-segment of small intestine as 5, ileum as 6, ascending colon as 7, descending colon as 8, and sigmoid as 9). We performed statistical analysis to investigate the correlation between the timing of ICG injection and the farthest segment of the digestive tract with fluorescence imaging.

### Preliminary exploration and application of ICG combined with near-infrared fluorescence imaging system in patients with abdominal adhesions

We included two cholecystectomized patients in our study, both of whom were females, aged 32 and 35, respectively. Patient A had previously undergone two surgeries due to bile duct stones. She was readmitted due to a recurrence of these stones, and the diagnosis of “bile duct stones” was confirmed, prompting a surgical treatment plan. Patient B had undergone cholecystectomy procedure three years ago due to gallstones and acute cholecystitis. She returned to the hospital after experiencing recurring abdominal pain over the past two weeks, and the diagnosis revealed the presence of residual gallstones. Considering the history of abdominal surgeries in both patients, we administered an indocyanine green (ICG) injection prior to surgery based on prior research findings. During the procedure, we utilized fluorescence laparoscopy to guide us in performing laparoscopic surgery on the patient.

### Statistical analysis

Fluorescence intensity was calculated using the IPP software, followed by fluorescence intensity ratios. The comparison of fluorescence intensity ratios between different wells was analyzed using an independent samples t-test. The correlation between ICG concentration and fluorescence imaging intensity, protein concentration and fluorescence imaging intensity, and the correlation analysis between ICG injection timing and fluorescence imaging in the farthest part of the digestive tract were conducted using Kendall’s tau-b correlation analysis. Statistical analysis was performed using SPSS software version 19.0 (SPSS Inc., Chicago, Illinois, USA).

### Supplementary Information

Supplementary Information 1.

Supplementary Video 1.

## Data Availability

The data sets used and/or analysed for the present study are not available owing to the limitations of ethical approvals. The analytical methods can be made available to other researchers on reasonable request to the corresponding author.
