# Supplementary material for: Real-time fluorescence-guided adhesiolysis with indocyanine green in intra-abdominal surgery (with video)
Source: Sci Rep. 2024 Jan 6;14:726. doi: 10.1038/s41598-024-51450-8 (PMC10771464; doi:10.1038/s41598-024-51450-8)
Supplement: Supplementary file 1 — Supplementary Information 1. [file 41598_2024_51450_MOESM1_ESM.docx]

Video title: Release of intra-abdominal adhesions using a near-infrared fluorescence imaging system in a cholecystectomized patient

Video legend: We conducted a choledochoscopy procedure on a 32-year-old female patient with abdominal adhesions. Preoperatively, 10 mg of indocyanine green was administered via peripheral venous injection 20 hours and 4 hours before surgery, respectively. Intraoperatively, we employed a near-infrared fluorescence imaging system to facilitate the dissection of adhesions.
